# Mutations based on viral decay acceleration in the HIV-1 genomes of a clinical population treated with the mutagenic nucleoside KP1461

**DOI:** 10.1186/1742-4690-8-S2-P84

**Published:** 2011-10-03

**Authors:** James I Mullins, Laura Heath, James P Hughes, Deyu Li, John M Essigmann, Kevin S Harris, Jeffrey H Simpson, Jean-Pierre Laurent, Lawrence A Loeb, Jeff Parkins

**Affiliations:** 1 Department of Microbiology, University of Washington, Seattle, WA USA; 2 Department of Medicine School of Medicine, University of Washington, Seattle, WA USA; 3 Department of Biostatistics School of Medicine, University of Washington, Seattle, WA USA; 4 Department of Pathology, University of Washington, School of Medicine, Seattle, WA USA; 5 Department of Chemistry Massachusetts Institute of Technology, Cambridge, MA USA; 6 Department of Biological Engineering, Massachusetts Institute of Technology, Cambridge, MA USA; 7 Koronis Pharmaceuticals, Redmond, WA USA

## Background

The deoxycytidine analog KP1212, and its prodrug KP1461, are prototypes of a new class of antiretroviral drugs designed to increase viral mutation rates, with the goal of eventually causing the collapse of the viral population. KP1212 will diminish or extinguish HIV-1 replication in cell culture, and has had very good safety profiles in clinical trials. Here we present an extensive analysis of viral sequences from patients from the first “mechanism validation” phase II clinical trial in which “salvage” patients received 1600 mg of drug twice per day for 124 days. The Phase IIa study did not demonstrate significant diminution of viral load. This was not unexpected as prior cell culture studies showed little change in viral titer prior to population collapse [1]. In the present work we sought to identify a subclinical impact of KP1461 therapy [2].

## Materials and methods

We performed direct gene sequencing of a very large number (>100) of sequences derived from individual HIV-1 RNA templates, after 0, 56 and 124 days of therapy from 10 treated and 10 untreated individuals. NMR studies were performed to identify tautomeric forms of the drug.

## Results

Private mutations, those not found in multiple viruses, were similar in treated and control individuals at day 0 (p=0.28), but were increased in treated individuals after 56 (p=0.02) and 124 (p=0.001) days of drug treatment. Furthermore, the spectrum of mutations observed in the treated group was distinct from that of the controls, with an excess of A to G and G to A mutations (p=0.01), and to a lesser extent T to C and C to T mutations (p=0.09), in the treated group. Tautomeric forms of the drug were detected that are predicted to result in a substantial number of misincorporation events during DNA synthesis.

## Conclusions

The predicted mechanism of action of the drug (transition mutations with a bias toward A<>G) was demonstrated *in vivo*. The observed increase in mutations in treated patients and the chemical properties of the new drug support a new mechanism of action by a novel antiretroviral therapy in humans.
